# Research trends on clinical fecal microbiota transplantation: A biliometric analysis from 2001 to 2021

**DOI:** 10.3389/fimmu.2022.991788

**Published:** 2022-10-21

**Authors:** Yu Li, Yaobin Ouyang, Cong He

**Affiliations:** ^1^Department of Gastroenterology, The first affiliated Hospital of Nanchang University, Nanchang, China; ^2^Department of Oncology, Mayo Clinic, Rochester, MN, United States

**Keywords:** fecal microbiota transplantation, research trends, CiteSpace, VOSviewer, bibliometric analysis

## Abstract

**Background:**

Numerous studies on fecal microbiota transplantation (FMT) have been conducted in the past two decades. We aimed to assess the research trends and hotspots in the field of FMT through a quantitative method.

**Materials and Methods:**

The clinical studies of FMT published from 2001 to 2021 were extracted from the Web of Science database. We analyzed the countries, institutions, authors, and keywords of these articles and visually illustrated using VOSviewer and CiteSpace software. The current application of FMT in clinical practice, including indications, efficacy, adverse events, as well as its methodology, such as donor, delivery route, were also evaluated.

**Results:**

A total of 227 records were finally identified. The number and rate of annual publications increased gradually. The USA ranked highest in the number of publications. Harvard University was the most influential institution, and Digestive Diseases and Sciences was the most productive journal. Kassam Zain published the most papers, and the high-frequency keywords were mainly related to diseases and techniques. Healthy donors were the most widely used donors, and frozen stool had the highest frequency of use. The predominant delivery route was endoscopy followed by oral capsules and enema. FMT was most frequently performed for the treatment of recurrent Clostridium Difficile Infection. The overall efficacy of FMT was 76.88%, and the incidence of minor and severe adverse events were 11.63% and 1.59%, respectively.

**Conclusions:**

This study delineated a comprehensive landscape of the advancement in FMT field. Although in its infancy, FMT is a burgeoning option for the treatment of a variety of diseases associated with gut dysbiosis. To improve the efficacy and reduce adverse events, future studies are warranted to optimize the methodology of FMT.

## Introduction

Fecal microbiota transplantation (FMT) is an innovative approach that transfers gut microbes from a healthy donor into the patient’s gastrointestinal tract to reconstitute the recipient’s gut homeostasis (1). The history of FMT dates back to the fourth century in China which was reported in the first Chinese handbook of emergency medicine, “Zhou Hou Bei Ji Fang” (or “Handy Therapy for Emergencies”) written up by Hong Ge (2). At that time, human fecal material, called yellow soup, was used in patients with severe diarrhea and achieved amazing efficacy. Then, Eiseman and his colleagues treated patients with pseudomembranous colitis successfully using FMT in 1958, and it was the first report on FMT in the medical literature (3). In 2013, the first randomized controlled trial was carried out by Els et al., which turned out that the infusion of donor feces was significantly more effective for the treatment of recurrent Clostridium difficile infection (rCDI) compared to the use of vancomycin (4). With the increasing understanding of gut microbiota in the development of human diseases, reports of FMT in microbiota-related disorders outside of Clostridium difficile infection (CDI) are emerging (5). Recently, a series of consensus has been published to standardize the clinical management of FMT, including donor screening, fecal material preparation, and delivery procedures (6–8). The different response of patients to FMT indicated that the composition of donor microbiota and the interplay between donor and recipient determine the efficacy of FMT. Therefore, it is critical to develop donor-recipient matching methods for the selection of suitable donors instead of super donors in order to improve efficacy and perform FMT precisely.

Bibliometric analysis is a quantitative analysis that helps to identify the current research characteristics and the research trends of a specific field by searching a literature database (9). Providing access to multiple databases and multidisciplinary content, the Web of Science database is often used for bibliometric analyses (10). On top of that, multiple analytical stools including VOSviewer software created by Nees Jan van Eck and Ludo Waltman and CiteSpace software created by Chaomei Chen are utilized to visualize the results of a bibliometric analysis, which offers a convenient way to describe researchers’ efforts in the investigation of a field (11, 12). So far, few bibliometric analyses have been examined on FMT with only one study extracted data from 2004 to 2017 (13).

FMT has been utilized sporadically in the early years as revealed by case reports and retrospective studies (14, 15). Subsequently, a great number of randomized clinical trials (RCTs) have been carried out to explore the efficacy and safety of FMT on a variety of diseases, including inflammatory bowel diseases (IBD), irritable bowel syndrome (IBS), metabolic disorders, etc (16, 17). Then, mounting reviews and meta-analyses are published to summarize the current findings on a specific point of this field (18–20). However, the quantitative study to comprehensively analyze the trajectory of this field is lacking. This bibliometric analysis is aimed to provide an overview of clinical FMT and elucidate its research trends and hotspots.

## Materials and methods

### Search strategy

Data were required from the Web of Science database *via* the Nanchang University Library website (http://lib.ncu.edu.cn/) for this bibliometric analysis. The searching used a combination of the following keywords and terms: [(Fecal OR Intestinal OR Gut OR Donor) AND (Microbiota OR Microbiome OR Microbe OR Bacterial OR Feces OR Microflora OR Flora) AND (Transplantation OR Transfer OR Transplant OR Infusion)] OR FMT OR Fecal-Transplantation OR Fecal-Transplant OR bacteriotherapy. The publication language was restricted to English, and the time span was from 2001 (January 01, 2001) to 2021 (December 31, 2021).

### Study selection

All the relevant publications were evaluated and collected by reading the titles and abstracts acquired from SCI-E of the Web of Science Core Collection (WoSCC) database. If necessary, the full text was downloaded from Web of Science or other databases. Articles were included according to the following criteria: (1) the study involved patients treated with FMT; (2) the main topic of the article was the application of FMT to a certain disease; (3) all types of clinical studies including retrospective studies, non-RCTs, RCTs and case series were analyzed. We excluded basic research, reviews, and case reports.

### Data extraction

A structured data extraction form was designed using Microsoft Excel 2019, which consisted of the following sections for analysis: (1) Publication information including title, author (first author and their corresponding country), study type, citation number of the publication, citations per publication (defined as total publication number/total publications), published journal, the journal impact factor (IF) of 2020 and H-index (defined as the number of papers with citation number > or = H) (21); (2) Disease or condition; (3) Methods and process of FMT; (4) Outcomes and overall conclusions including the efficacy of treatment, authors’ conclusions (effective, ineffective or unclear) and adverse events.

### Data analysis

After extraction, data analysis was performed using VOSviewer software (version 1.6.18), CiteSpace (version 6.1.R1), and GraphPad (version 9). Visual cooperation network graphs of authors, institutions, countries, and keywords were generated by VOSviewer. Each term (author, institution, country, and keyword) was indicated using a circle. The distance between two circles varied according to the strength of the link between terms. Different clusters were represented by different colors. The size of circles was positively correlated with the appearance frequency of terms, and the thickness of the line indicated the strength of the connection between terms (22). CiteSpace software was utilized to visualize the research trends of keywords. It mainly focused on identifying the time, frequencies, and centralities of keywords co-occurrence networks. GraphPad software was used to perform other bibliometric analyses.

## Results

### Study selection and characteristics

**Figure 1:**
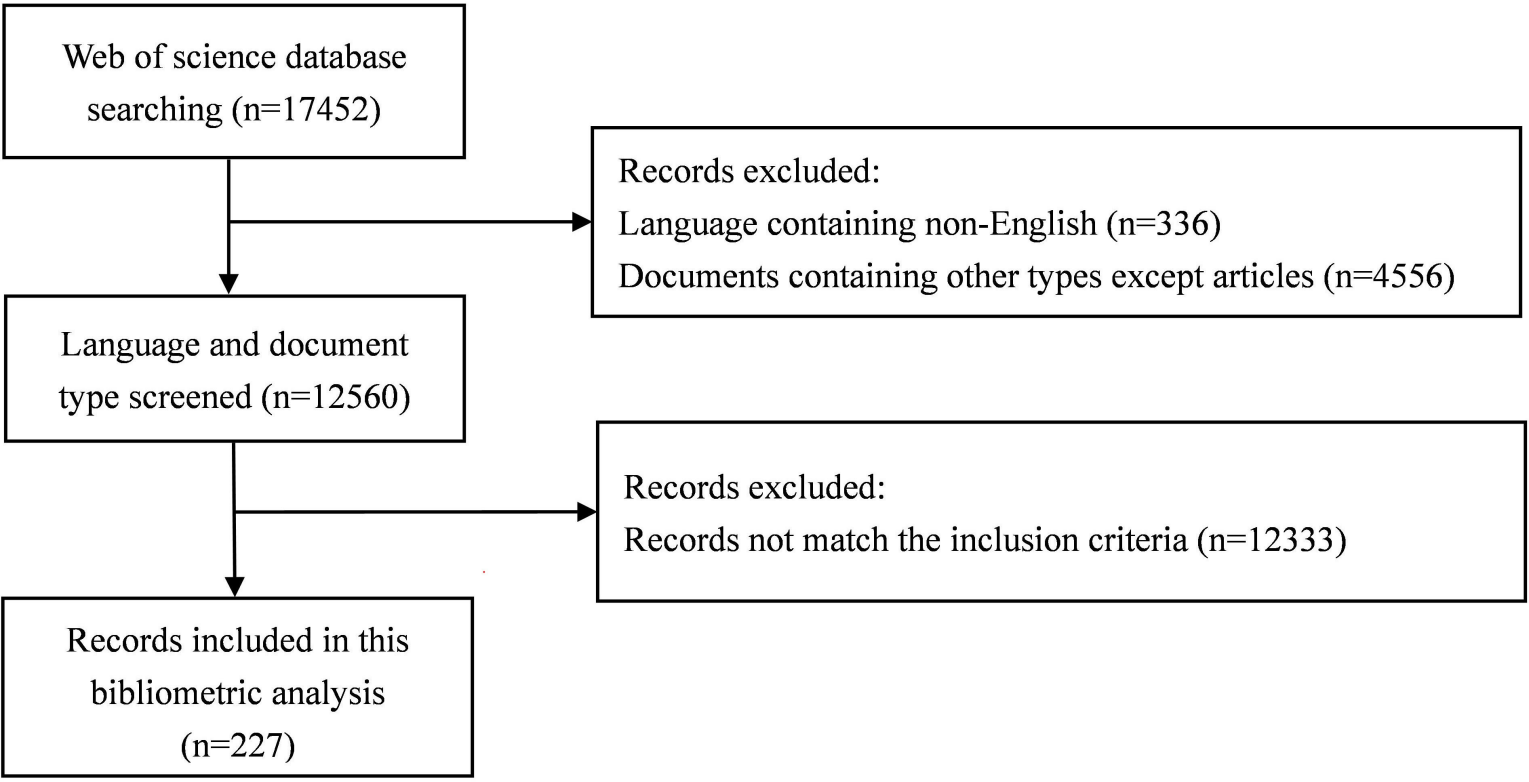
The flowchart of the literature screening process.

From 2001 to 2021, a total of 17452 publications were identified through our search strategy in WoSCC. 4892 publications were excluded because they are not articles and non-English language. The titles and abstracts of the remaining 12560 publications were carefully evaluated according to our inclusion criteria. As a result, 227 articles were included in the analysis (**Figure 1**).

**Figure 2:**
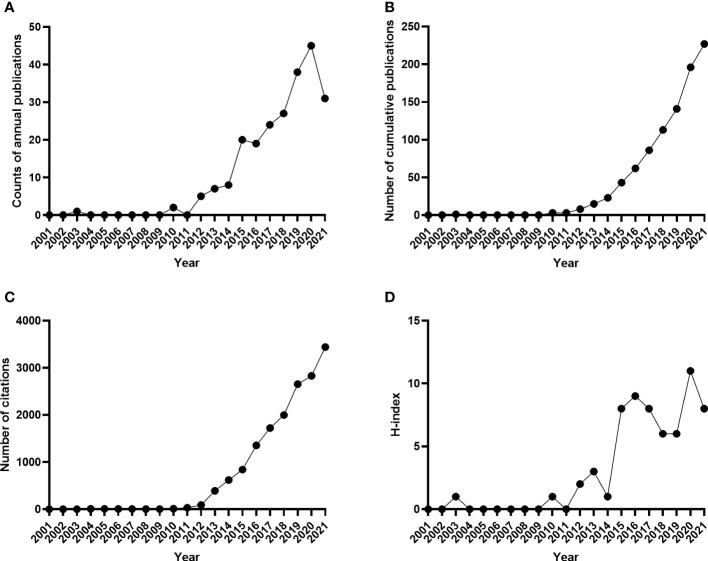
Trends in the number of publications from 2001-2021 in the field of FMT. **(A)** The annual number of published articles; **(B)** The number of annual cumulative articles; **(C)** The annual citation number of publications; **(D)** The annual H-index values of publications.

The annual number of publications on FMT is presented in **Figure 2A**. Before 2011, there were few studies in this field. One article was published in 2003 (n=1), and it was in 2012 that the number of publications began to increase gradually. The peak of annual publications was reached in 2020, which accounts for 19.82% of all publications. Although the number of publications decreased slightly in 2016 and 2021, there were increasing trends in cumulative publications (**Figure 2B**). The annual citations were low from 2001 to 2013 (below 500 annual citations), increased rapidly since 2014 and then remained at a high number (more than 1000 annual citations) after 2016 (**Figure 2C**). While the annual H-index was extremely low (≤2) before 2015, it elevated up to 6 or more from then on (**Figure 2D**). There were still decreasing fluctuations during 2016-2018 and 2020-2021.

To analyze the quality of selected studies, we classified articles into RCTs and non-RCTs. Since the first RCT published in 2013, the overall number of prospective RCTs increased to ten publications annually from 2019 till now (**Figure S1**), indicating that the quality of enrolled research was rising prominently in recent years. **Table 1** presents the details of the 10 most cited studies, and six of them were RCTs. They were mainly focused on the application of FMT on CDI, especially in rCDI, as well as Ulcerative Colitis (UC).

**Table 1:** The top 10 most cited articles in the field of clinical fecal microbiota transplantation from 2001 to 2021.

Rank	Author	Journal	Title	Citations of Web of Science	Institution
1	Els van Nood et al.	N Engl J Med. 2013 Jan 31;368 (5):407-15	Duodenal Infusion of Donor Feces for Recurrent Clostridium difficile	2183	University of Amsterdam
2	Paul Moayyedi et al.	Gastroenterology. 2015 Jul;149 (1):102-109.e6	Fecal Microbiota Transplantation Induces Remission in Patients With Active Ulcerative Colitis in a Randomized Controlled Trial	782	McMaster University
3	Sudarshan Paramsothy et al.	Lancet. 2017 Mar 25;389 (10075):1218-1228	Multidonor intensive faecal microbiota transplantation for active ulcerative colitis: a randomised placebo-controlled trial	568	St Vincent’s Hospital
4	Noortje G Rossen et al.	Gastroenterology. 2015 Jul;149 (1):110-118.e4	Findings From a Randomized Controlled Trial of Fecal Transplantation for Patients With Ulcerative Colitis	512	University of Amsterdam
5	Lawrence J Brandt et al.	Am J Gastroenterol. 2012 Jul;107 (7):1079-87	Long-Term Follow-Up of Colonoscopic Fecal Microbiota Transplant for Recurrent Clostridium difficile Infection	450	Montefiore Medical Center
6	Matthew J Hamilton et al.	Am J Gastroenterol. 2012 May;107 (5):761-7	Standardized Frozen Preparation for Transplantation of Fecal Microbiota for Recurrent Clostridium difficile Infection	445	University of Minnesota
7	Ilan Youngster et al.	JAMA. 2014 Nov 5;312 (17):1772-8	Oral, Capsulized, Frozen Fecal Microbiota Transplantation for Relapsing Clostridium difficile Infection	413	Boston Children’s Hospital
8	Colleen R Kelly et al.	Am J Gastroenterol. 2014 Jul;109 (7):1065-71	Fecal Microbiota Transplant for Treatment of Clostridium difficile Infection in Immunocompromised Patients	400	Women’s Medicine Collaborative, Alpert Medical School of Brown University
9	Christine H Lee et al.	JAMA. 2016 Jan 12;315 (2):142-9	Frozen vs Fresh Fecal Microbiota Transplantation and Clinical Resolution of Diarrhea in Patients With Recurrent Clostridium difficile Infection A Randomized Clinical Trial	379	St Joseph’s Healthcare Hamilton
10	G Cammarota et al.	Aliment Pharmacol Ther. 2015 May;41 (9):835-43	Randomised clinical trial: faecal microbiota transplantation by colonoscopy vs. vancomycin for the treatment of recurrent Clostridium difficile infection	346	Institute of Internal Medicine, Catholic University

### Country, institution, author, keyword, and journal analysis

**Figure 3:**
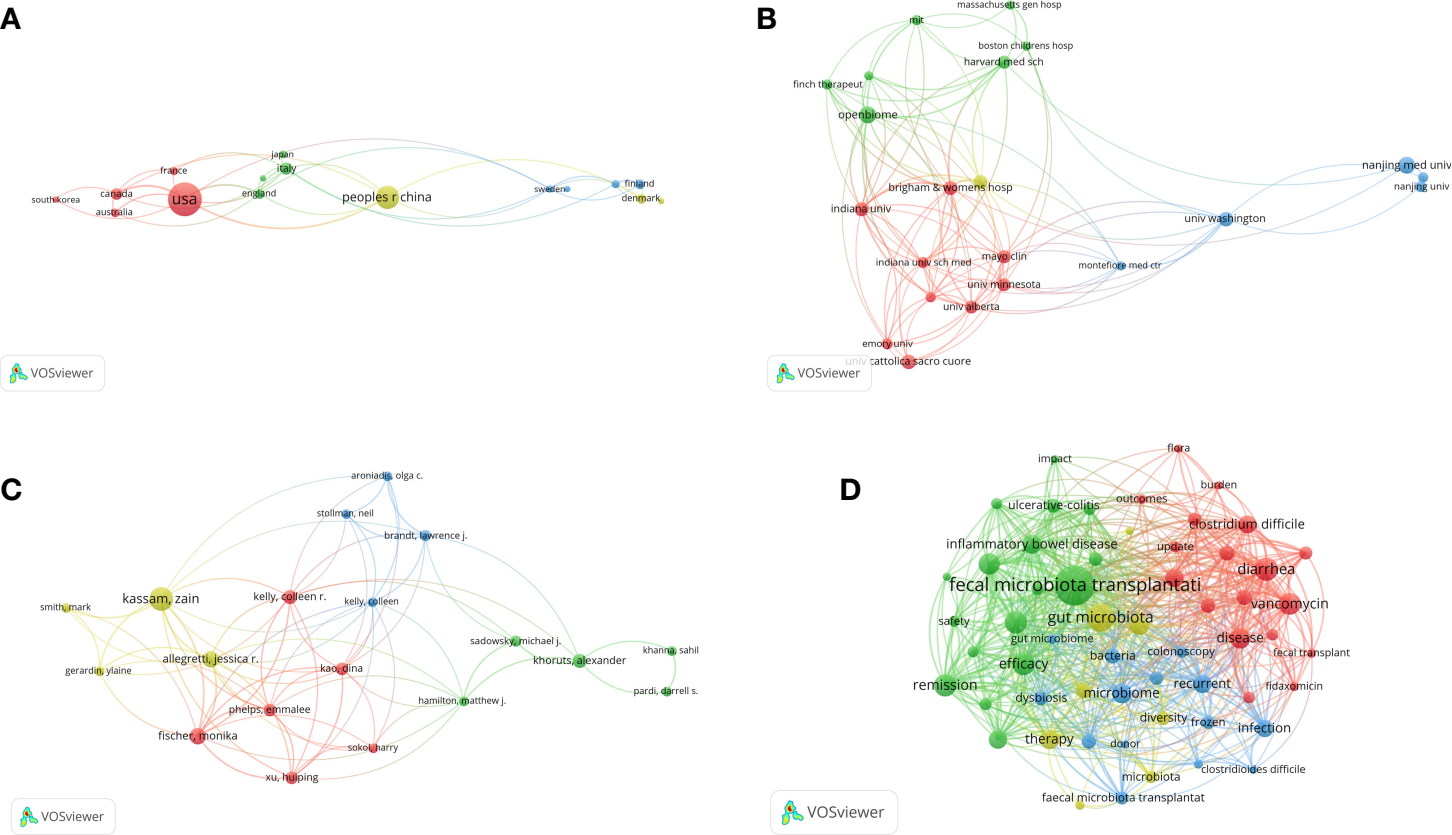
The cooperation network of countries/regions **(A)**, institutions **(B)**, and scholars **(C)** in the field of FMT. The network visualization of the keywords **(D)**. The nodes denoted elements such as countries/regions, institutions, authors, or keywords. Larger nodes represented higher number of publications or occurrences, and the more links among different nodes indicated the stronger correlations.

The international cooperation of different countries is shown in **Figure 3A**. A total of 16 countries/regions and 63 co-operations are displayed. The USA was the country that most frequently participated in international cooperation, followed by Canada, England, China and Italy. USA not only collaborated with European countries, such as England, France, Sweden, but also collaborated with Asian countries, including Japan, China, South Korea. Then we analyzed the H-index, total citations, citations per publication of the 10 most productive countries/regions (**Table S1**). The greatest number of publications came from the USA (n=91), followed by China (n=46), Italy (n=15), and Canada (n=14). While the USA owned the largest number of H-index (44) and Citations (7700), the Netherlands had the largest number of citations per publication (357.5), whose total publications and citations were 8 and 2860, respectively. Besides, USA was also the most productive countries followed by China, with the annual publications increased dramatically in the past decade (**Figure S2**).

A total of 22 institutions and 213 co-operations are displayed in **Figure 3B**. Next, we analyzed the H-index, total citations, and citations per publication of the 10 most productive institutions (**Table S2**). Harvard University had the largest number of H-index (13) and citations (1760), and Brown University had the highest citations per publication (144.25), suggesting their leading status in FMT.

**Figure 4:**
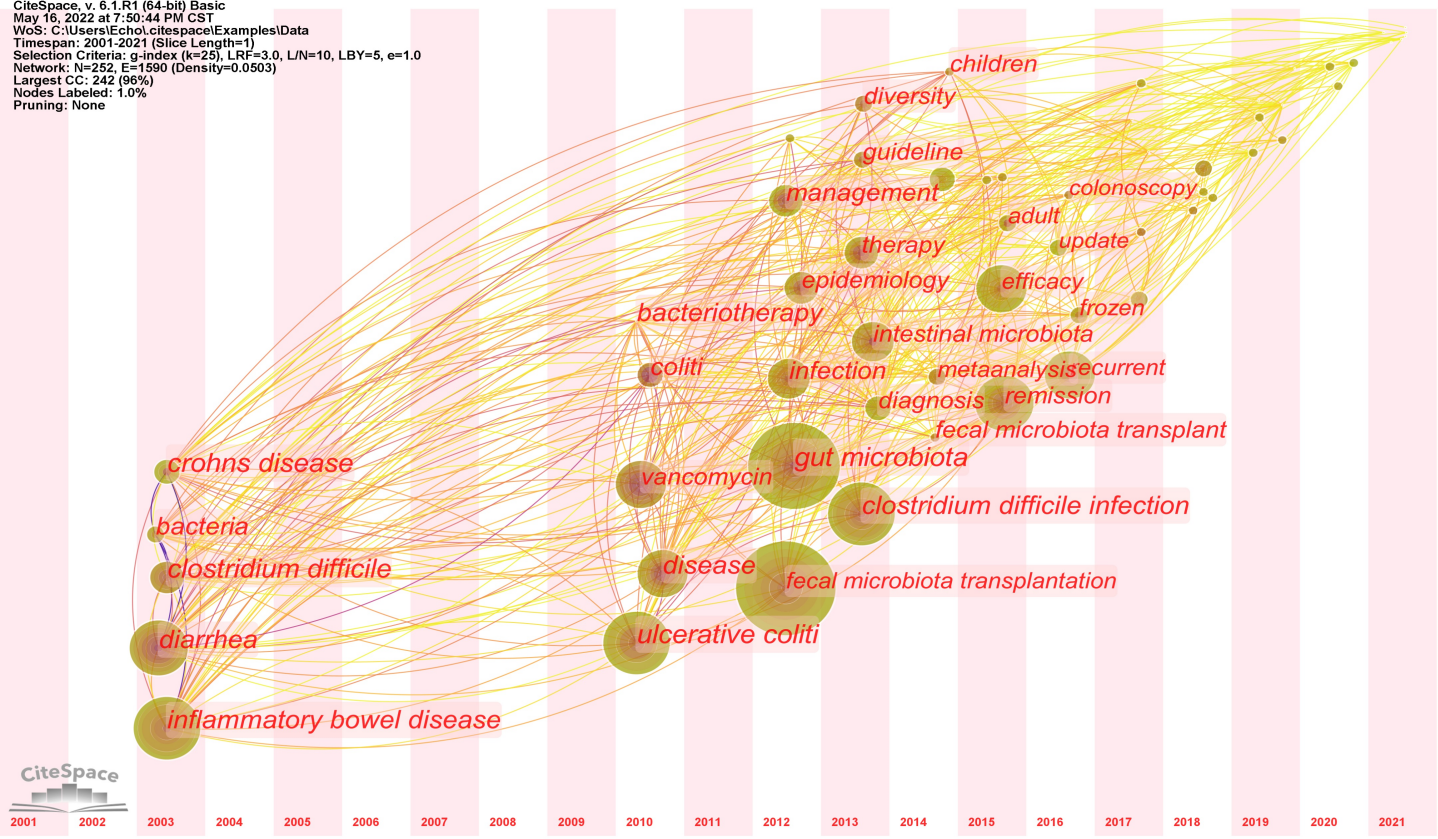
Chronological variation of the keywords related to FMT from 2001 to 2021. The size of each dot represented the frequency of keywords occurrence, while the links indicated the co-occurrence of keywords.

There were more than 1500 authors contributing to the enrolled publications. The network map of authors produced by VOSviewer was presented in **Figure 3C**. A total of 19 authors were displayed. Kassam Zain had the greatest number of articles (n=16), followed by Zhang Faming (n=12), Cui Bota (n=11), and Li Ning (n=10). The visualization map of keywords that appeared more than eight times was shown in **Figure 3D**. While some of the keywords were disease-related, including “clostridium difficile”, “inflammatory bowel disease”, “efficacy”, “safety”, others were associated with operational protocols, such as “donor”, “colonoscopy”, “frozen”. Moreover, the time zone map of keywords showed that “diarrhea”, “inflammatory bowel disease” and “clostridium difficile” were first identified in 2003, and then “recurrent clostridium difficile infection” as well as the formalization of clinical practice of FMT, including “guideline”, “management”, have been increasingly focused (**Figure 4**).

The 227 articles were published in 112 journals. We collected the total citations, citations per publication, H-index, and journal IF (2020) of the most productive 10 journals (**Table S3**). Digestive Diseases and Sciences (n=12), Alimentary Pharmacology Therapeutics (n=11), and Inflammatory Bowel Diseases (n=10) were the most productive 3 journals. Among the most productive 10 journals, Gastroenterology had the highest journal IF (2020) (22.682). American Journal of Gastroenterology had both the highest citations (1758) and citations per publication (251.14).

### Research focus analysis

#### Analysis of donor selection

**Figure 5:**
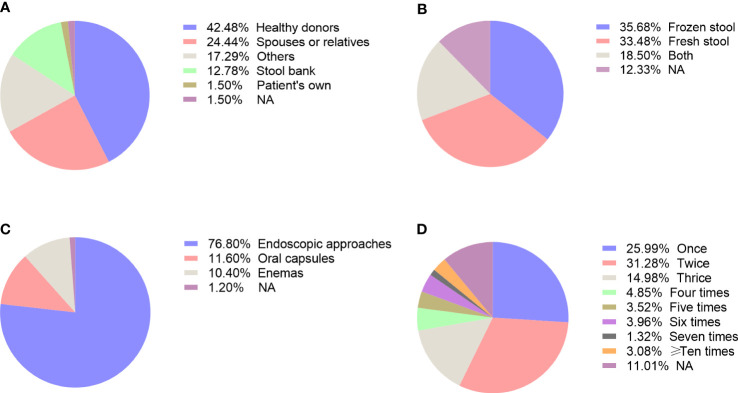
The methodology issues related to FMT, including choice of donor **(A)**, fecal preparation **(B)**, delivery routes **(C)**, and frequency of administration **(D)**.

As shown in **Figure 5A**, donors for FMT were mainly from healthy individuals (42.48%), followed by spouses or relatives (24.44%), others (17.29%), stool bank (12.78%), and patient’s own (1.50%). Others included close friends, partners, patient-selected donors, etc. In addition, we defined NA as unclear donor sources in the articles.

#### Analysis of stool preparation

**Figure 5B** showed the fecal material in the enrolled studies, which can be divided into the frozen stool, fresh stool, both of them, and NA. It can be clearly seen that frozen stool had the highest proportion of usage (n=81, 35.68%). Except for 28 studies (12.33%) that did not figure out the formulation of stool, 76 studies (33.48%) used fresh stool and 42 studies (18.50%) used both the frozen stool and fresh stool.

#### Analysis of delivery routes

The main delivery routes of FMT included endoscopic tubes, oral capsules, and enemas. More than half of the studies performed FMT *via* endoscopic tubes (76.80%) (**Figure 5C**). Besides, the proportion of using oral capsules and enemas was 11.60% and 10.40% respectively, while others did not point out the exact mode of delivery (1.20%). Among the endoscopic routes, a majority of studies infused the fecal suspension through colonoscopy that accounts for 61.46%, while the upper gastrointestinal routes involved nasojejunal tube (14.06%), gastroscope (10.94%), nasogastric tube (9.90%), and nasoduodenal tube (6.25%).

#### Analysis of administration frequency

The frequency of FMT varied among different studies. Over 50% of studies performed FMT less than three times, with 25.99% articles reported a single infusion, 31.28% twice and 14.98% thrice (**Figure 5D**). There were 7 articles (3.08%) even administered 10 times or more FMT. Meanwhile, the time interval of each FMT ranged from 12 hours to more than 3 years, which varied from study to study even for the same disease. So far, there is limited guidance to standardize the number and frequency of FMT for clinical practice.

#### Analysis of indications

**Figure 6:**
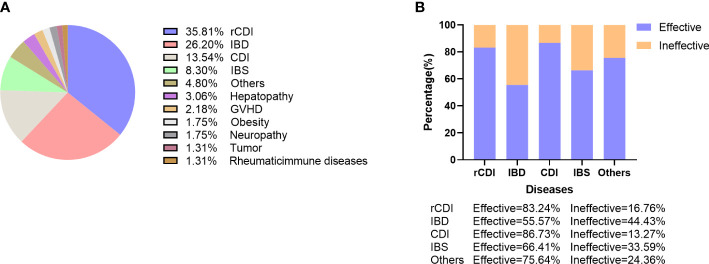
The indications of FMT and its potential efficacy. **(A)** Proportion of studies that used FMT in different diseases. **(B)** Overall efficacy of FMT on the top 5 most common diseases.

As shown in **Figure 6A**, FMT has been applied in a wide range of diseases, including not only intestinal diseases but also metabolic, neural disorders as well as cancer. The most common disease using FMT was CDI (13.54%), especially rCDI (35.81%), followed by IBD (26.20%), and IBS (8.30%). Among patients with IBD, studies of FMT on UC (n=41) were much more than that on Crohn’s Disease (CD) (n=11). Given the increasing recognition of gut-liver and gut-brain axis, FMT has also been administered in hepatic diseases, including hepatic encephalopathy (n=2), cirrhosis (n=2), steatohepatitis (n=1), nonalcoholic fatty liver (n=1) and chronic hepatitis B (n=1), as well as neurological disorders, including Parkinson’s disease (n=2), Autism Spectum Disorder (n=1) and Tourette Syndrome (n=1). The application of FMT on Graft-versus-host disease (n=5), rheumatic immune diseases (n=3) and PD-1 assisted antitumor immunotherapy (n=3) further demonstrated the role of gut microbiota on the regulation of host immunity. Then the distribution of disease spectrum was analyzed in the 5 most productive countries. As shown in the **Figure S3**, USA, Italy, Canada, and Denmark mainly focused on rCDI, while China had the highest proportion of clinical trials on IBD.

#### Analysis of efficacy and safety

The overall efficacy of FMT in enrolled studies was 76.88%. Among the top 5 diseases, CDI had the highest efficacy (86.73%), followed by rCDI (83.24%), IBS (66.41%), and IBD (55.57%). Among patients with IBS, Functional Constipation had the highest efficacy (81.16%), followed by Functional Diarrhea (70.59%) and Slow Transit Constipation (50.62%). The role of FMT in IBD was not as efficacious as expected, with similar efficacy between CD (59.90%) and UC (56.81%). However, the efficacy of FMT on refractory CD (76.67%) appeared higher than refractory UC (36.17%). Overall, about three quarters of the studies (76.65%) reported positive results of FMT as a therapeutic option while it was ineffective in 5.73% studies (**Figure 6B**). Taken together, in addition to the highly effective role of FMT in CDI, its efficacy for treating other diseases has been modest with a much higher variability in patient response.

The safety of FMT is assessed by the occurrence of adverse events (AEs), including mild or moderate adverse events (MAEs) and severe adverse events (SAEs). According to the statistics we collected, the overall frequency of MAEs and SAEs was 11.63% and 1.59%, respectively. The most frequently reported MAEs was comprised of diarrhea, abdominal pain, flatulence, bloating, which were self-limiting. On the whole, FMT seems to be a safety procedure under standard protocol and stringent supervision.

## Discussion

Since the successful application of fecal microbiota transplantation (FMT) on CDI in 2013, both clinicals and scientists have focused on FMT as a new method to restore gut microbiota homeostasis and translate from bench to bedside (4, 23). In this bibliometric analysis, we evaluated 227 clinical studies of FMT after searching the Web of Science database and manually screening eligible articles. Our study revealed that the annual number of publications and H-index gradually increased from 2012 and reached the peak in 2020. On the subject of the citations in this field, there was a strong breakthrough (over 1000) in 2016 and after that the annual number of citations increased rapidly, indicating that FMT has gained great interest and broad acceptance in the past few years. Moreover, a series of consensus on the regulation, production and use of FMT in clinical practice has been released successively from different countries (6–8).

First of all, we analyzed the most influential countries, institutions, and journals in the research of FMT. The USA was the most cooperative country and owned the highest number of publications, H-index values, and citations, while the Netherlands had the largest number of citations per publication, which suggest that USA and Netherlands were the most influential countries in FMT application. Furthermore, we found that the publications of the USA followed by China increased most quickly in the past 5 years, showing their great potential in this field. Besides, Harvard University was the most productive institution, with the highest H-index values and total citations, and Brown University had the highest citations per publication. With respect to journals that published FMT studies, our data suggested that Digestive Diseases and Sciences had the largest number of publications, and Alimentary Pharmacology Therapeutics had the highest H-index values. The journal with the most citations was American Journal of Gastroenterology, while Gastroenterology had the largest number of citations per publication and the highest 2020 Journal IF.

The efficacy and safety of FMT has been investigated in a wide range of disorders that are associated with gut microbiota dysbiosis. We found that CDI, especially rCDI, was the most common disease using FMT, which accounted for nearly 50%. A large body of evidence, including randomized clinical trials, systematic reviews and meta-analyses, has clearly proven that FMT is a highly effective approach for rCDI, which has been enrolled in global guidelines with strong recommendation (6, 8, 24). Additionally, the role of FMT has been extensively explored in two other enteric diseases, such as IBD and IBS, yet the outcome is inconsistency. Although FMT is efficacious as induction therapy in mild to moderate UC, it has not been recommended as first-line therapy. Given the fewer clinical trials of FMT on CD and IBS compared to UC, there is insufficient evidence for FMT in the treatment of these two intestinal disorders. Of note, recent studies have reported that FMT exhibited promising effects on inducing high response rates of patients with IBS and maintaining remission in CD patients (25, 26). Besides, there are mounting studies exploring the impact of FMT on extra-intestinal diseases, including metabolic disorders, hepatic diseases, graft-versus-host disease, neurological disorders and tumor (5). Although researchers observed that FMT induced favorable changes of microbiota and clinical improvement of these diseases, its optimal place in the current therapeutic algorithm is needed to be defined (27). Consistent with previous studies, our study showed that the incidence of FMT-related adverse events was low with most of them being mild and self-limiting (28). Although FMT seems to be safety, it is vital to improve the methodology and strengthen the supervision of FMT, especially during the pandemic of corona virus disease (COVID-19).

The methodology of FMT is a crucial determinant for the successful colonization of healthy microbiota in the gut of patients. Thus, we analyzed the potential factors that influence the efficacy of FMT, including donor selection, preparation of fecal material, routes of administration, and the frequency of transplantation. Approximately half of the enrolled studies employed healthy donors to provide fecal microbiota while 24.4% from spouses or relatives. Compared to manually prepared fecal microbiota, an automatic extraction and purification system for washed microbiota transplantation has been reported to be safer, more precise and more quality-controllable (29). The stool bank, such as OpenBiome, is an alternative for hospitals without preparation facility as it provides reliable, convenient and safety access to FMT. We found that frozen stool has been chosen as much as fresh stool. Although a decline in microbial viability has been reported in frozen stool compared to fresh stool, their efficacy was comparable in clinical resolution of patients with rCDI (30). With the development of encapsulation technique, the application of oral capsule will on the rise in the future as it not only achieved similar efficacy to colonoscopy delivery in treating rCDI but also noninvasive, convenient and esthetic to patients (31). No matter the fecal material used in FMT is fresh or frozen, it is vital to choose the optimal donors that largely determines the success rate of FMT. Current screening criteria of donors mainly focus on biosafety issues that ensure to prevent any transmissible risk factors, such as pathogens or diseases. However, the healthy gut microbiome from those donors might not be suitable for all patients with different microbiota. The concept of “super-donors” has been emerged as patients received their fecal materials exhibited higher remission rate, which might attribute to their diversified gut microbiota (32). Since it is difficult to find the super-donors, donor-recipient matching for FMT based on machine learning methods will be helpful for rational donor selection and the performance of precise FMT in the coming future (33).

Delivery methods can be broadly classified into upper and lower gastrointestinal routes. We observed that colonoscopy accounted for more than half of the studies, which was more commonly used compared to other delivery routes. Evidence from the comparisons among different routes of FMT largely indicate equal efficacy between upper and lower gastrointestinal methods (34). Additionally, our study showed that a majority of trials performed less than 3 times of transplantation, although there is no agreed standard for administration frequency. Several researches even conducted more than 10 times of FMTs according to the response of the patients (35).

There also exist several limitations to our study. First, our publications were only derived from SCI-E of the WoSCC database, which may lead to the result of incomplete literature. Other databases such as PubMed and Scopus may produce slightly different results. Nevertheless, the Web of Science database is the most popular and widely recognized database for bibliometric analyses (36). Second, we excluded non-English publications and may not take eligible articles published in other languages into consideration. Third, some bias during the selection of publications may not be avoided, although two people were assigned to review the initial results.

In conclusion, this study summarized the trends and hotspots of FMT during the past two decades. The annual publications of FMT are increased rapidly and will continue to grow in the years to come. While FMT has been widely recommended for the treatment of CDI and rCDI, its application has been extended to other gastrointestinal as well as extra-gastrointestinal diseases although mixed efficacy results are reported in different clinical trials, most likely due to sample size, research design and methodology. Further optimization of FMT protocols, such as super-donor characterization and capsule-based delivery, will help to boost its clinical success and become a patient-friendly strategy for disease management.

## Data availability statement

The original contributions presented in the study are included in the article/**Supplementary Material**. Further inquiries can be directed to the corresponding author.

## Author contributions

YL performed the literature search, data extraction, and wrote the manuscript. CH and YO designed the study and revised the manuscript. YL and YO performed the statistical analysis and contributed equally as co-first authors. All authors contributed to the article and approved the submitted version.

## Funding

This work was supported by the National Natural Science Foundation of China (no. 81860106), grants from the Science and Technology Department of Jiangxi Province (no. 20204BCJL23048).

## Conflict of interest

The authors declare that the research was conducted in the absence of any commercial or financial relationships that could be construed as a potential conflict of interest.

## Publisher’s note

All claims expressed in this article are solely those of the authors and do not necessarily represent those of their affiliated organizations, or those of the publisher, the editors and the reviewers. Any product that may be evaluated in this article, or claim that may be made by its manufacturer, is not guaranteed or endorsed by the publisher.

## References

[1] Gupta A Khanna S. Fecal microbiota transplantation. JAMA (2017) 318(1):102–. doi: 10.1001/jama.2017.6466 28672320

[2] Zhang F Luo W Shi Y Fan Z Ji G. Should we standardize the 1,700-year-old fecal microbiota transplantation? Am J Gastroenterol (2012) 107(11):1755. doi: 10.1038/ajg.2012.251 23160295

[3] Eiseman B Silen W Bascom GS Kauvar AJ. Fecal enema as an adjunct in the treatment of pseudomembranous enterocolitis. Surgery (1958) 44(5):854–9. 13592638

[4] van Nood E Vrieze A Nieuwdorp M Fuentes S Zoetendal EG de Vos WM. Duodenal infusion of donor feces for recurrent clostridium difficile. N Engl J Med (2013) 368(5):407–15. doi: 10.1056/NEJMoa1205037 23323867

[5] Ooijevaar RE Terveer EM Verspaget HW Kuijper EJ Keller JJ. Clinical application and potential of fecal microbiota transplantation. Annu Rev Med (2019) 70:335–51. doi: 10.1146/annurev-med-111717-122956 30403550

[6] Haifer C Kelly CR Paramsothy S Andresen D Papanicolas LE McKew GL. Australian Consensus statements for the regulation, production and use of faecal microbiota transplantation in clinical practice. Gut (2020) 69(5):801–10. doi: 10.1136/gutjnl-2019-320260 32047093

[7] Ng SC Kamm MA Yeoh YK Chan PKS Zuo T Tang W. Scientific frontiers in faecal microbiota transplantation: Joint document of Asia-pacific association of gastroenterology (APAGE) and Asia-pacific society for digestive endoscopy (APSDE). Gut (2020) 69(1):83–91. doi: 10.1136/gutjnl-2019-319407 31611298 PMC6943253

[8] Cammarota G Ianiro G Tilg H Rajilic-Stojanovic M Kump P Satokari R. European Consensus conference on faecal microbiota transplantation in clinical practice. Gut (2017) 66(4):569–80. doi: 10.1136/gutjnl-2016-313017 PMC5529972 28087657

[9] Farias da Cruz M Baraúna Magno M Alves Jural L Pimentel TC Ferreira DMTP Esmerino EA. Probiotics and dairy products in dentistry: A bibliometric and critical review of randomized clinical trials. Food Res Int (2022) 157:111228. doi: 10.1016/j.foodres.2022.111228 35761544

[10] Shen L Wang S Dai W Zhang Z. Detecting the interdisciplinary nature and topic hotspots of robotics in surgery: Social network analysis and bibliometric study. J Med Internet Res (2019) 21(3):e12625. doi: 10.2196/12625 30912752 PMC6454338

[11] van Eck NJ Waltman L. Software survey: VOSviewer, a computer program for bibliometric mapping. Scientometrics (2010) 84(2):523–38. doi: 10.1007/s11192-009-0146-3 PMC2883932 20585380

[12] Synnestvedt MB Chen C Holmes JH. CiteSpace II: visualization and knowledge discovery in bibliographic databases. AMIA Annu Symp Proc (2005) 2005:724–8. PMC1560567 16779135

[13] Li Y Zou Z Bian X Huang Y Wang Y Yang C. Fecal microbiota transplantation research output from 2004 to 2017: A bibliometric analysis. PeerJ (2019) 7:e6411. doi: 10.7717/peerj.6411 30809438 PMC6387576

[14] Borody TJ Warren EF Leis S Surace R Ashman O. Treatment of ulcerative colitis using fecal bacteriotherapy. J Clin Gastroenterol (2003) 37(1):42–7. doi: 10.1097/00004836-200307000-00012 12811208

[15] Brandt LJ Aroniadis OC Mellow M Kanatzar A Kelly C Park T. Long-term follow-up of colonoscopic fecal microbiota transplant for recurrent clostridium difficile infection. Am J Gastroenterol (2012) 107(7):1079–87. doi: 10.1038/ajg.2012.60 22450732

[16] Camacho-Ortiz A Gutierrez-Delgado EM Garcia-Mazcorro JF Mendoza-Olazaran S Martinez-Melendez A Palau-Davila L. Randomized clinical trial to evaluate the effect of fecal microbiota transplant for initial clostridium difficile infection in intestinal microbiome. PloS One (2017) 12(12):19. doi: 10.1371/journal.pone.0189768 PMC5738078 29261736

[17] Karjalainen EK Renkonen-Sinisalo L Satokari R Mustonen H Ristimaki A Arkkila P. Fecal microbiota transplantation in chronic pouchitis: A randomized, parallel, double-blinded clinical trial. Inflammation Bowel Dis (2021) 27(11):1766–72. doi: 10.1093/ibd/izab001 PMC8528148 33501942

[18] Crowther M Lim W Crowther MA. Systematic review and meta-analysis methodology. Blood (2010) 116(17):3140–6. doi: 10.1182/blood-2010-05-280883 20656933

[19] Green JE Davis JA Berk M Hair C Loughman A Castle D. Efficacy and safety of fecal microbiota transplantation for the treatment of diseases other than clostridium difficile infection: A systematic review and meta-analysis. Gut Microbes (2020) 12(1):1–25. doi: 10.1080/19490976.2020.1854640 PMC7757860 33345703

[20] Xu D Chen VL Steiner CA Berinstein JA Eswaran S Waljee AK. Efficacy of fecal microbiota transplantation in irritable bowel syndrome: A systematic review and meta-analysis. Am J Gastroenterol (2019) 114(7):1043–50. doi: 10.14309/ajg.0000000000000198 PMC7257434 30908299

[21] Hirsch JE. An index to quantify an individual's scientific research output. Proc Natl Acad Sci U S A. (2005) 102(46):16569–72. doi: 10.1073/pnas.0507655102 PMC1283832 16275915

[22] Perianes-Rodriguez A Waltman L van Eck NJ. Constructing bibliometric networks: A comparison between full and fractional counting. J Informetr (2016) 10(4):1178–95. doi: 10.1016/j.joi.2016.10.006

[23] Kassam Z Lee CH Yuan Y Hunt RH. Fecal microbiota transplantation for clostridium difficile infection: Systematic review and meta-analysis. Am J Gastroenterol (2013) 108(4):500–8. doi: 10.1038/ajg.2013.59 23511459

[24] Cammarota G Ianiro G Kelly CR Mullish BH Allegretti JR Kassam Z. International consensus conference on stool banking for faecal microbiota transplantation in clinical practice. Gut (2019) 68(12):2111–21. doi: 10.1136/gutjnl-2019-319548 PMC6872442 31563878

[25] El-Salhy M Winkel R Casen C Hausken T Gilja OH Hatlebakk JG. Efficacy of fecal microbiota transplantation for patients with irritable bowel syndrome at three years after transplantation. Neurogastroenterol Motil (2022) 34(1):e14200. doi: 10.1111/nmo.14200 34145677

[26] Kong L Lloyd-Price J Vatanen T Seksik P Beaugerie L Simon T. Linking strain engraftment in fecal microbiota transplantation with maintenance of remission in crohn's disease. Gastroenterology (2020) 159(6):2193–202 e5. doi: 10.1053/j.gastro.2020.08.045 32860788 PMC7725862

[27] D'Haens GR Jobin C. Fecal microbial transplantation for diseases beyond recurrent clostridium difficile infection. Gastroenterology (2019) 157(3):624–36. doi: 10.1053/j.gastro.2019.04.053 PMC7179251 31220424

[28] Marcella C Cui B Kelly CR Ianiro G Cammarota G Zhang F. Systematic review: the global incidence of faecal microbiota transplantation-related adverse events from 2000 to 2020. Aliment Pharmacol Ther (2021) 53(1):33–42. doi: 10.1111/apt.16148 33159374

[29] Zhang T Lu G Zhao Z Liu Y Shen Q Li P. Washed microbiota transplantation vs. manual fecal microbiota transplantation: Clinical findings, animal studies and *in vitro* screening. Protein Cell (2020) 11(4):251–66. doi: 10.1007/s13238-019-00684-8 PMC7093410 31919742

[30] Lee CH Steiner T Petrof EO Smieja M Roscoe D Nematallah A. Frozen vs fresh fecal microbiota transplantation and clinical resolution of diarrhea in patients with recurrent clostridium difficile infection: A randomized clinical trial. Jama (2016) 315(2):142–9. doi: 10.1001/jama.2015.18098 26757463

[31] Halaweish HF Boatman S Staley C. Encapsulated fecal microbiota transplantation: Development, efficacy, and clinical application. Front Cell Infect Microbiol (2022) 12:826114. doi: 10.3389/fcimb.2022.826114 35372103 PMC8968856

[32] Wilson BC Vatanen T Cutfield WS O'Sullivan JM. The super-donor phenomenon in fecal microbiota transplantation. Front Cell Infect Microbiol (2019) 9:2. doi: 10.3389/fcimb.2019.00002 30719428 PMC6348388

[33] He R Li P Wang J Cui B Zhang F Zhao F. The interplay of gut microbiota between donors and recipients determines the efficacy of fecal microbiota transplantation. Gut Microbes (2022) 14(1):2100197. doi: 10.1080/19490976.2022.2100197 35854629 PMC9302524

[34] Ramai D Zakhia K Ofosu A Ofori E Reddy M. Fecal microbiota transplantation: donor relation, fresh or frozen, delivery methods, cost-effectiveness. Ann Gastroenterol (2019) 32(1):30–8. doi: 10.20524/aog.2018.0328 PMC6302197 30598589

[35] Paramsothy S Kamm MA Kaakoush NO Walsh AJ van den Bogaerde J Samuel D. Multidonor intensive faecal microbiota transplantation for active ulcerative colitis: a randomised placebo-controlled trial. Lancet (2017) 389(10075):1218–28. doi: 10.1016/S0140-6736(17)30182-4 28214091

[36] Ma D Yang B Guan B Song L Liu Q Fan Y. A bibliometric analysis of pyroptosis from 2001 to 2021. Front Immunol (2021) 12:731933. doi: 10.3389/fimmu.2021.731933 34484243 PMC8416445

