# Supplementary figures and images for: Research trends on clinical fecal microbiota transplantation: A biliometric analysis from 2001 to 2021
Source: Front Immunol. 2022 Oct 21;13:991788. doi: 10.3389/fimmu.2022.991788 (PMC9639330; doi:10.3389/fimmu.2022.991788)

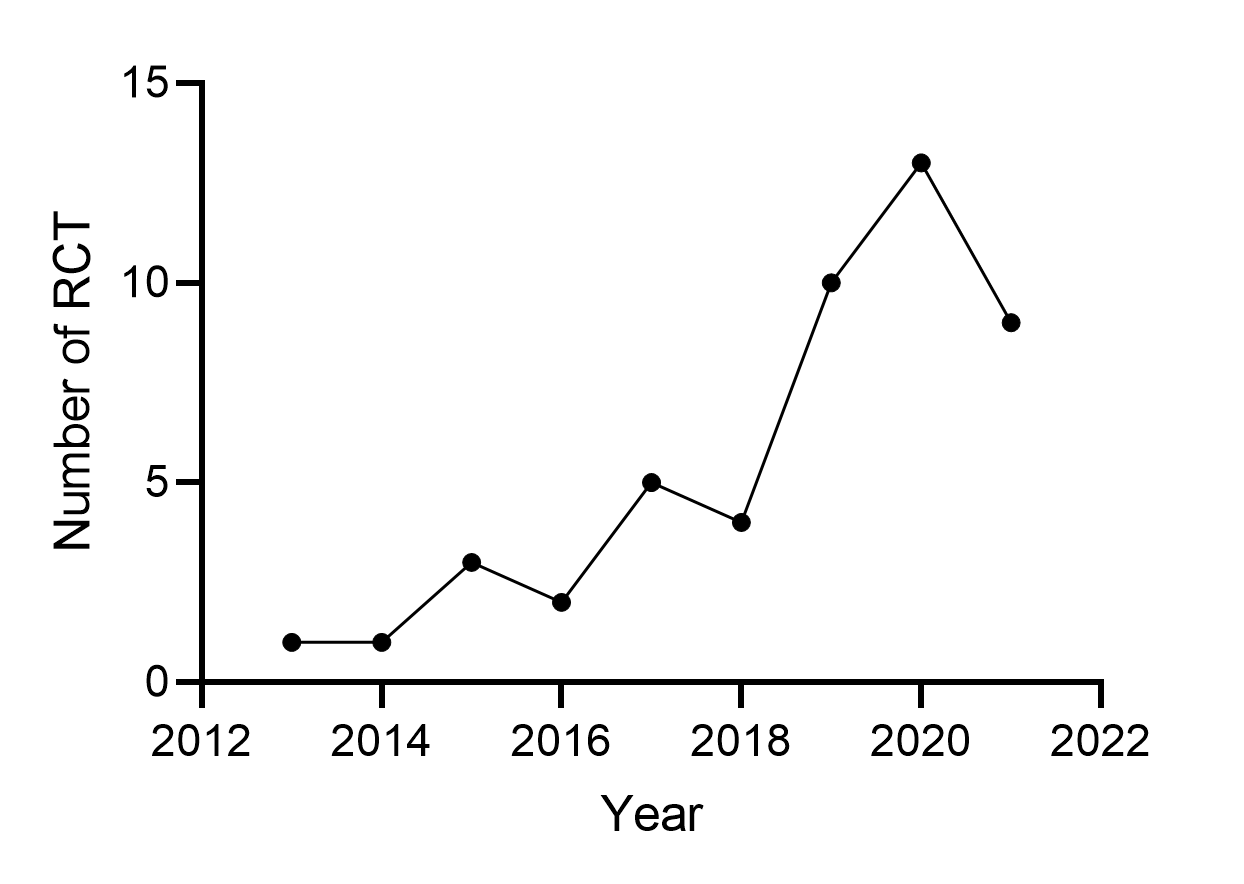

Supplement: Supplementary file 1 [file Image_1.tif]

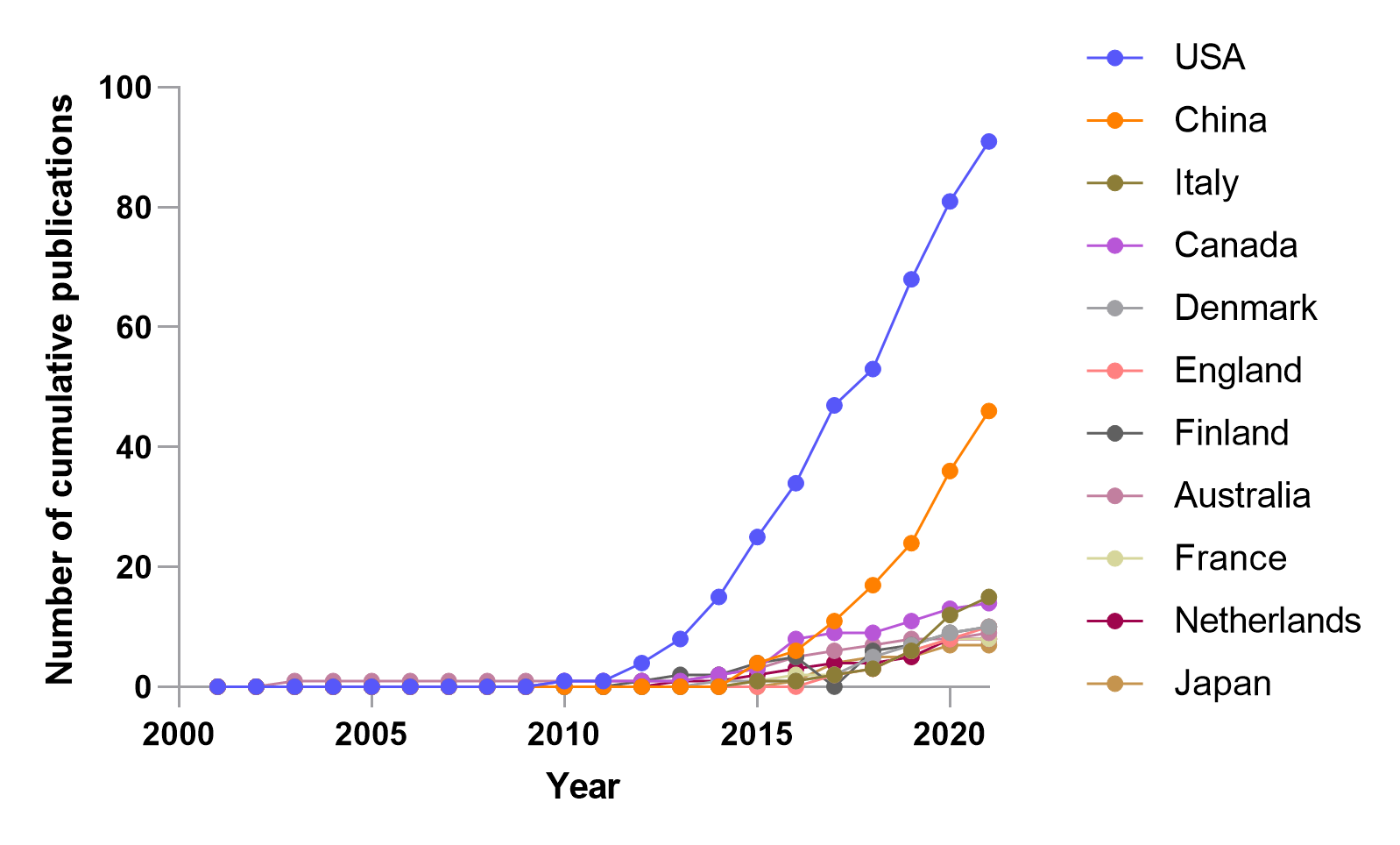

Supplement: Supplementary file 2 [file Image_2.tif]

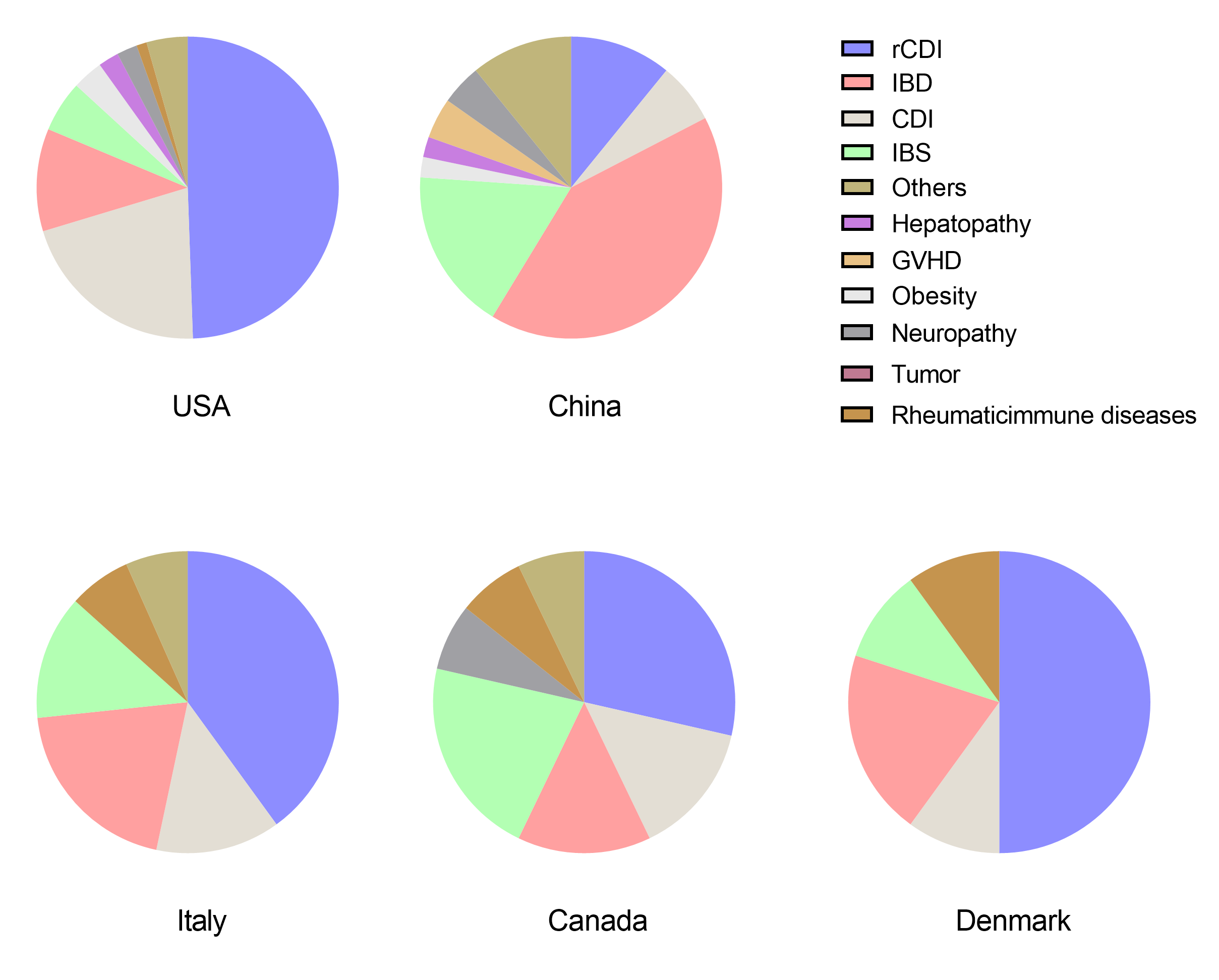

Supplement: Supplementary file 3 [file Image_3.tif]
