# Supplementary material for: Research trends on clinical fecal microbiota transplantation: A biliometric analysis from 2001 to 2021
Source: Front Immunol. 2022 Oct 21;13:991788. doi: 10.3389/fimmu.2022.991788 (PMC9639330; doi:10.3389/fimmu.2022.991788)
Supplement: Supplementary file 4 [file Table_1.docx]

Table S1: The top 10 most productive countries/regions in the field of clinical fecal microbiota transplantation from 2001 to 2021.

| Countries/Regions | Publications | H-index | Citations | Citations per-publication |
| --- | --- | --- | --- | --- |
| USA | 91 | 44 | 7700 | 84.62 |
| China | 46 | 21 | 1654 | 35.96 |
| Italy | 15 | 9 | 658 | 43.87 |
| Canada | 14 | 11 | 2507 | 179.07 |
| Denmark | 10 | 8 | 542 | 54.2 |
| England | 10 | 8 | 474 | 47.4 |
| Finland | 10 | 8 | 3158 | 315.8 |
| Australia | 9 | 7 | 1774 | 197.11 |
| France | 8 | 7 | 375 | 46.88 |
| Netherlands | 8 | 7 | 2860 | 357.5 |
| Japan | 7 | 6 | 437 | 62.43 |
