# Supplementary material for: Research trends on clinical fecal microbiota transplantation: A biliometric analysis from 2001 to 2021
Source: Front Immunol. 2022 Oct 21;13:991788. doi: 10.3389/fimmu.2022.991788 (PMC9639330; doi:10.3389/fimmu.2022.991788)
Supplement: Supplementary file 5 [file Table_2.docx]

Table S2: The top 10 most productive institutions in the field of clinical fecal microbiota transplantation from 2001 to 2021.

| Institutions | Publications | H-index | Citations | Citations per-publication |
| --- | --- | --- | --- | --- |
| Harvard University | 23 | 13 | 1760 | 76.52 |
| Indiana University System | 14 | 11 | 1223 | 87.36 |
| Nanjing Medical University | 14 | 11 | 598 | 42.71 |
| Openbiome | 14 | 11 | 779 | 55.64 |
| Brigham Women’s Hospital | 12 | 10 | 378 | 31.5 |
| Brown University | 12 | 10 | 1731 | 144.25 |
| Catholic University of the Sacred Heart | 11 | 7 | 588 | 53.45 |
| Irccs Policlinico Gemelli | 11 | 7 | 588 | 53.45 |
| University of Washington | 11 | 11 | 1536 | 139.64 |
| University of Washington Seattle | 11 | 11 | 1536 | 139.64 |
