# Supplementary material for: Research trends on clinical fecal microbiota transplantation: A biliometric analysis from 2001 to 2021
Source: Front Immunol. 2022 Oct 21;13:991788. doi: 10.3389/fimmu.2022.991788 (PMC9639330; doi:10.3389/fimmu.2022.991788)
Supplement: Supplementary file 6 [file Table_3.docx]

Table S3: The top 10 leading journals in the field of clinical fecal microbiota transplantation from 2001-2021.

| Journal | Publications | Citations | Citations per-publication | H-index | Journal IF(2020) |
| --- | --- | --- | --- | --- | --- |
| *Digestive Diseases and Sciences* | 12 | 241 | 20.08 | 9 | 3.199 |
| *Alimentary Pharmacology Therapeutics* | 11 | 1022 | 92.91 | 10 | 8.171 |
| *Inflammatory Bowel Diseases* | 10 | 635 | 63.5 | 9 | 5.325 |
| *American Journal of Gastroenterology* | 7 | 1758 | 251.14 | 7 | 10.864 |
| *Journal of Clinical Gastroenterology* | 7 | 747 | 106.71 | 7 | 3.062 |
| *Clinical Gastroenterology and Hepatology* | 6 | 322 | 53.67 | 6 | 11.382 |
| *Plos One* | 6 | 297 | 49.5 | 6 | 3.24 |
| *BMC Gastroenterology* | 5 | 40 | 8 | 3 | 3.067 |
| *Clinical Infectious Diseases* | 5 | 667 | 133.4 | 5 | 9.079 |
| *Gastroenterology* | 5 | 1653 | 330.6 | 5 | 22.682 |
| *Journal of Pediatric Gastroenterology and Nutrition* | 5 | 360 | 72 | 5 | 2.839 |
